# Gradenigo’s Syndrome and Vernet Syndrome as Presenting Signs of Nasopharyngeal Carcinoma

**DOI:** 10.7759/cureus.41636

**Published:** 2023-07-10

**Authors:** Ciji Robinson, Diva Maraj, Jasdeep S Minhas, Mehakmeet Bhatia, Vivek Kak

**Affiliations:** 1 Internal Medicine, Henry Ford Health System, Jackson, USA; 2 Medicine, St. George's University School of Medicine, St. George's, GRD; 3 Infectious Disease, Henry Ford Health System, Jackson, USA

**Keywords:** methicillin resistant staphylococcus aureus (mrsa), mrsa, nasopharyngeal carcinoma, vernet syndrome, gradenigo's syndrome

## Abstract

Both Gradenigo’s syndrome and Vernet syndrome are rare pathologies of the intracranial space; both involve compression of a particular anatomic location in the skull, thus affecting structures nearby or within that space. A patient presenting with one or both of these syndromes should raise concern for malignancy, head trauma, or an intracranial infection. We present a case of a 39-year-old female with three weeks of left-sided ear, face, and neck pain along with difficulty swallowing and reduced vision in the left eye. Magnetic resonance imaging of the brain revealed fullness in the left nasopharyngeal region, raising concern for malignancy or infection. Biopsy of the mass ultimately revealed Epstein-Barr virus positive nasopharyngeal carcinoma, nonkeratinizing undifferentiated type, along with culture data revealing methicillin-resistant *Staphylococcus aureus* positive left otomastoiditis. She received chemoradiation therapy along with six weeks of antibiotic therapy. A patient presenting with symptoms reflective of a sinus infection unrelieved by antibiotics with concomitant cranial nerve deficits should raise clinical concern for an intracranial pathology rather than a simple case of sinusitis.

## Introduction

Multiple cranial neuropathies is a term used to describe the many foci within the skull base containing nerves that can become compressed and subsequently cause neurologic deficits [1,2]. Some examples are trigeminal neuralgia, hemifacial spasm, and glossopharyngeal neuralgia [2]. Gradenigo’s syndrome involves infection of the petrous temporal bone at the skull base [3]. Cranial nerves (CNs) V and VI run together as they pass over the petrous temporal bone and thus become simultaneously affected in cases of Gradenigo’s syndrome [3]. The classic triad of Gradenigo’s syndrome is CN VI palsy, pain in the distribution of CN V, and suppurative otitis media [3], which results from obstruction at the Eustachian tube leading to a purulent infection. Vernet syndrome also presents as a triad involving CNs IX, X, and XI [4]. Vernet syndrome is characterized by compression of the jugular foramen by a mass (such as malignancy) or infection, and thus the three aforementioned nerves that traverse this foramen become compromised [4]. The jugular foramen is formed by both the petrous temporal bone and the occipital bone. Seeing how both Gradenigo’s syndrome and Vernet syndrome involve the petrous temporal bone as part of their anatomy, the two syndromes can present together [5]. We present a case of concomitant Gradenigo’s syndrome and Vernet syndrome in a patient who presented to the hospital for ear, face, and neck pain.

## Case presentation

**Figure 1:**
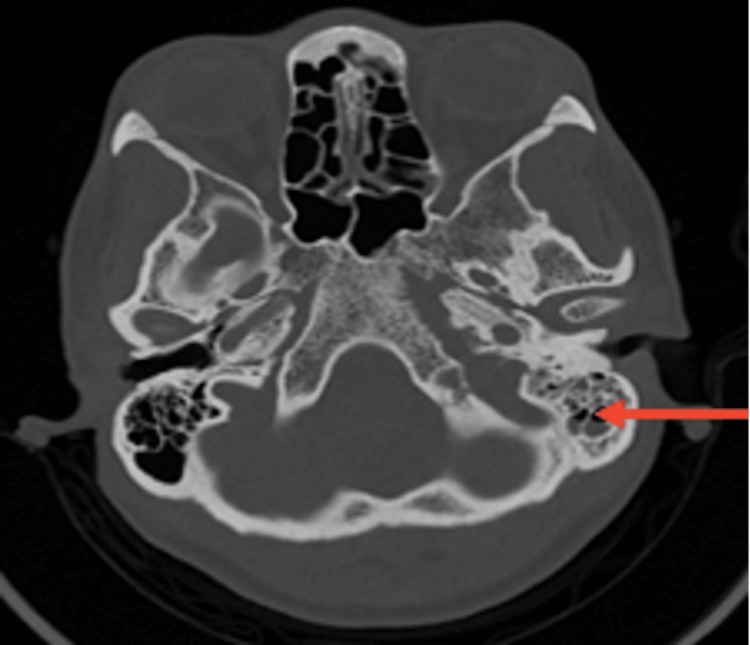
Head CT (axial plane, without contrast) revealing a left-sided otomastoiditis (arrow) CT, computed tomography

**Figure 2:**
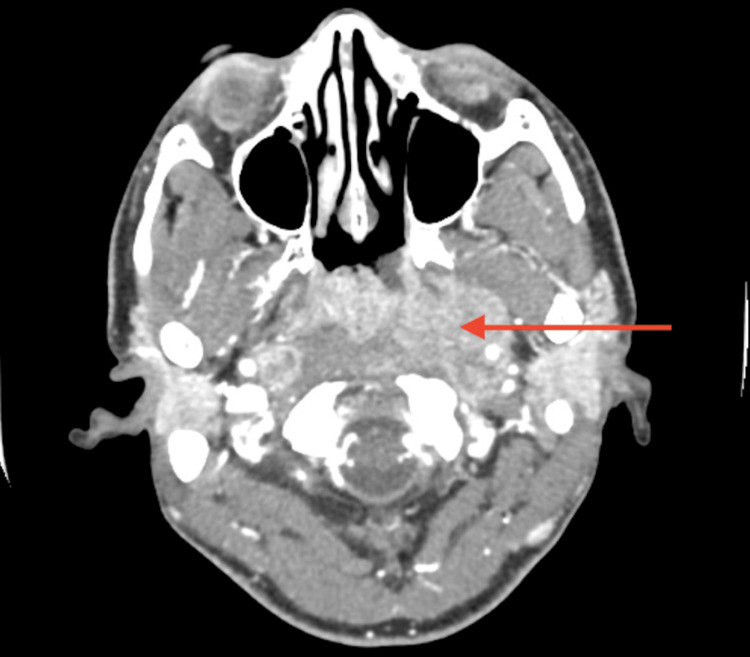
Head CT (axial plane, without contrast) revealing a left-sided nasopharyngeal soft tissue mass (arrow) CT, computed tomography

**Figure 3:**
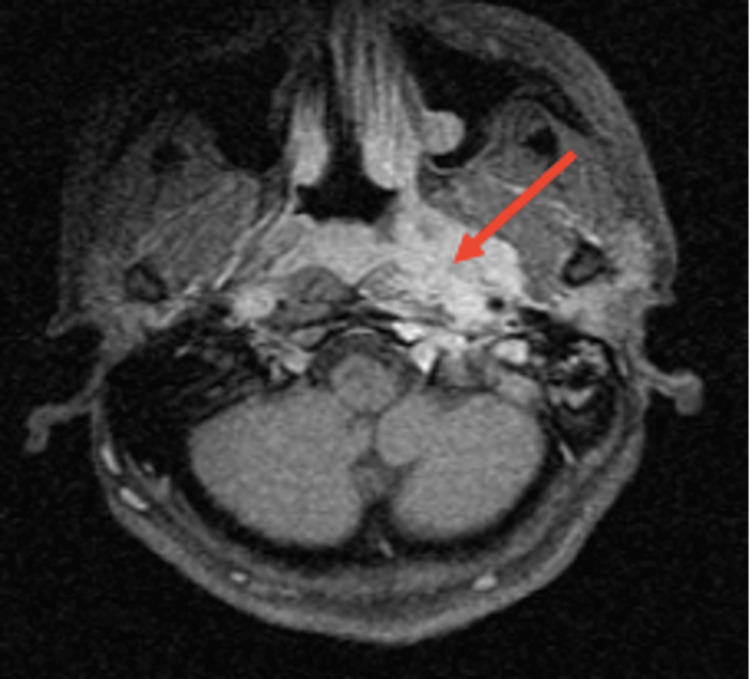
Brain MRI (axial plane, with and without 0.5 mmol/mL gadolinium) revealing a left-sided nasopharyngeal mass (arrow) MRI, magnetic resonance imaging

We present a case of a 39-year-old female African American with a past medical history of hypertension and tobacco dependence presenting to the emergency department with three weeks of left-sided ear, face, and neck pain. She stated that at the time of symptom onset, she was diagnosed with sinusitis and otitis media and was prescribed amoxicillin, which did not ultimately resolve her symptoms. She was hemodynamically stable upon presentation with all vital signs within normal limits, and physical examination was remarkable for severe left-sided facial hyperesthesia, left CN VI palsy, dysphagia (difficulty swallowing), voice hoarseness, and decreased strength with lifting of both shoulders upward against resistance. The remainder of her CN testing was normal, reflexes were 2+, and strength and sensation were both intact. She did not display uvular deviation from the midline. Her basic lab work including complete blood count, basic metabolic profile, and lactate were within normal limits aside from mild leukopenia of 3.7 K/uL. Computed tomography (CT) of the head was remarkable for left-sided otomastoiditis (Figure 1), along with an infiltrating, enhancing soft tissue mass involving the left lateral posterior nasopharynx (Figure 2). Subsequent magnetic resonance imaging (MRI) of the brain revealed an area of fullness in the left nasopharyngeal region, raising concern for an infectious or malignant mass (Figure 3). Our patient underwent biopsy of the nasopharyngeal mass via nasal endoscopy, and a fluid sample was also collected for culture and analysis. The pathology report revealed Epstein-Barr virus (EBV) positive nasopharyngeal carcinoma, nonkeratinizing undifferentiated type. She was also found to have pulmonary nodules on CT imaging of the chest as well as surrounding lymph node involvement and thus was staged as IVA nasopharyngeal carcinoma. Culture results of surrounding fluid also revealed growth of methicillin-resistant *Staphylococcus aureus*. Human immunodeficiency virus testing in our patient was negative.

Once diagnosed with nasopharyngeal carcinoma, our patient was started on a chemotherapeutic regimen of gemcitabine and cisplatin, as well as radiation therapy, and surgical intervention was not ultimately pursued. She tolerated and adhered to these treatments well and had a positive response on a follow-up positron emission tomography scan. She was maintained on a six-week antibiotic regimen following hospital discharge for otomastoiditis treatment, which initially consisted of intravenous vancomycin and ceftriaxone and was eventually converted to oral doxycycline and amoxicillin-clavulanic acid upon hospital discharge.

## Discussion

Gradenigo’s syndrome was first described in 1907 as a sequelae of bacterial suppurative otitis media that spreads into the intracranial space [5]. As a sequelae, CNs V and VI will become affected, thus leading to inability to abduct the eye on the affected side, as well as severe facial pain on the same side. A mass located in the intracranial space that is proximal to the Eustachian tube can eventually lead to infection and otitis media, thus mimicking the triad of Gradenigo’s syndrome [5]. Obstruction at this location leads to inability of the Eustachian tube to properly drain any infectious agents and thus subsequent suppurative otitis media can develop, thus completing the triad of Gradenigo’s syndrome [6]. The infection can also spread to other surrounding structures and cause otomastoiditis, as our patient had. In cases of simple otitis media, the most commonly implicated organisms in the adult population are *Streptococcus pneumoniae*, *Haemophilus influenzae*, and *Moraxella catarrhalis* [7]. Similarly, some commonly implicated organisms in cases of Gradenigo’s syndrome include *Staphylococcus* species, *Streptococcus* species, *Haemophilus influenzae*, *Pseudomonas aeruginosa*, and *Moraxella catarrhalis*; thus, initial antibiotics used to treat otitis media should cover these organisms [3].

Vernet syndrome can be seen in the setting of infection, such as varicella-zoster virus or herpes simplex virus; however, malignant, vascular, or traumatic etiologies can also be seen, with the most common cause being brain metastasis [4,8]. Our patient was diagnosed with Vernet syndrome given voice hoarseness, dysphagia, and dysfunction involving CN XI (weakness with shoulder abduction). Vernet syndrome can be seen as a sequela of Gradenigo’s syndrome as a mass can easily extend to the jugular foramen from the petrous temporal bone [5], which was the case for our patient. It is therefore evident that our patient’s nasopharyngeal mass significantly compressed both the petrous temporal bone and the jugular foramen as they are anatomically in close proximity within the skull base, thus leading to both syndromes presenting at once.

Common presenting symptoms of nasopharyngeal carcinoma involve nasal and otologic symptoms [9], both of which our patient had. Neurologic involvement due to extension of the tumor into the surrounding brain parenchyma can also be seen less commonly, including CN involvement [10].

## Conclusions

Gradenigo’s syndrome and Vernet syndrome presenting together should raise clinical suspicion for an underlying intracranial mass causing obstruction. It is imperative to obtain appropriate intracranial imaging in these cases to help delineate the underlying etiology. A patient presenting with typical symptoms of suppurative otitis media along with associated CN deficits should prompt further diagnostic work-up, as the patient likely has more than a simple case of otitis media or sinusitis.
